# Developing A Sustainable Urban-Environmental Quality Evaluation System in China Based on A Hybrid Model

**DOI:** 10.3390/ijerph16081434

**Published:** 2019-04-22

**Authors:** Qigan Shao, Sung-Shun Weng, James J.H. Liou, Huai-Wei Lo, Hongbo Jiang

**Affiliations:** 1 School of Economics & Management, Xiamen University of Technology, Xiamen 361024, China; qgshao@xmut.edu.cn (Q.S.); hbjiang@xmut.edu.cn (H.J.); 2 Graduate Institute of Industrial and Business Management, National Taipei University of Technology, Taipei 10608, Taiwan; w110168888@gmail.com; 3 Department of Information and Finance Management, National Taipei University of Technology, Taipei 10608, Taiwan; wengss@ntut.edu.tw; 4 Department of Industrial Engineering and Management, National Taipei University of Technology, Taipei 10608, Taiwan

**Keywords:** sustainable environmental quality, multiple attribute decision-making, fuzzy best worst method, grey relational analysis

## Abstract

In China, with the acceleration of urbanization, people pay more attention to the quality of urban environment. Air pollution, vegetation destruction, water waste and pollution, and waste sorting have restricted the sustainable development of urban environment. It is important to evaluate the impact of these environmental concerns as a prerequisite to implement an effective urban environmental sustainability policy. The aim of this paper is to establish a system for evaluating sustainable urban environmental quality in China. We extracted six dimensions and 29 criteria for assessing urban sustainable environment. Then, a fuzzy technique and the best worst method were applied to obtain the weights for the dimensions and criteria. Next, grey possibility values were applied to evaluate the sustainable environmental quality of five cities: Beijing, Shanghai, Shenzhen, Guangzhou, and Hangzhou in China. A sensitivity analysis was performed to identify how the ranking of these five cities changed when varying the weights of each criterion. The results show that pollution control, the natural environment, and water management are the three most important dimensions for urban environmental quality evaluation. We suggest that controlling pollutant emissions, strengthening food waste management, improving clean production processes, and utilizing heat energy are the effective measures to improve the urban environment and achieve sustainable urban environmental development.

## 1. Introduction

Under the background of economic growth and urbanization process in China, the urban environment has been disrupted, and the sustainable development of urban environment has been threatened [1]. For example, the urban heat island effect and the health problem of urban residents are the challenges for facing the sustainable development of cities [2]. The urban population in China has increased too rapidly and they are increasingly devoting their attention to ecological environment construction. Rapid urbanization and modernization have increased the pressure of urban environmental development in China. As the largest developing country in the world, China has made a rapid urbanization and modernization. China’s urbanization rate has increased from less than 20% in 1978 to 59.58% in 2018. However, the problem of environmental pollution has become increasingly prominent, such as China is one of the largest CO_2_ emitters, and China’s cities are under pressure to reduce energy consumption and emissions [3]. Concrete environmental construction measures have thus been proposed in China, where the government is promoting the ideas of an ecological civilization and devoting more effort toward ecological environmental protection. The Chinese government has always attached importance to the prevention of urban environment damage. Some measures such as garbage sorting, shared bicycles, and wastewater treatment have been proposed [4,5]. However, with rapid urban population growth, the urban environment is facing unprecedented challenges, such as the aggravation of pollution, resource shortages, and traffic congestion [6]. Moreover, cites have become a major source of CO_2_ emissions, accounting for approximately 70% of all such emissions [7].

Worsening of the urban metabolism and its impact on climate change pose challenges to the sustainable development of urban environments [8]. Hence, the following issues affecting the sustainable development of urban environments are worthy of attention. Firstly, which indicators affect the development of urban sustainable environment, and which of these are key indicators? Secondly, how should the quality of a sustainable environment in a city be evaluated through indicators? Finally, how can improve a city’s environment quality through its status quo? It is particularly essential to develop a sustainable urban environmental quality evaluation system that can serve as a reference for the construction of a sustainable city environment.

The aim of this paper is the development of a sustainable urban-environmental quality evaluation system in order to solve the above problems. The concept of sustainability mainly involves sustainable development and sustainable assessment [9]. Researchers have already made some contributions to the study of sustainable urban environments. Ahvenniemi et al. [10] proposed critical indicators for the sustainable development of a city environment and established a framework for its evaluation. Aldairi and Tawalbeh [11] provided a detailed overview of the main security problems in sustainable cities and their current solutions. They also proposed key indicators for the evaluation of intelligent urban environments. Alencar et al. [12] noted that natural resources, artificial environments, and energy management are the three pillars of sustainable environmental development. Although these studies have proposed some essential factors that influence environmental quality, specific indicators such as water management or pollution control have rarely been considered. Thus, evaluating the sustainable quality framework of a city is a multi-dimensional challenge involving water resources, environmental pollution, energy management, and other factors. Because it is difficult to collect actual data, this study uses the multiple attribute decision-making (MADM) method to evaluate environmental quality. MADM models are proposed to evaluate sustainable urban environmental quality and to help decision makers make sound decisions with limited information. Obviously, this paper mainly deals with the sustainability assessment problems, and sustainability assessment is related to the strong and weak sustainability paradigms. Ziemba (2019) argued that compensatory methods are used to solve the problem of weak sustainability, while the non-compensation method is suitable for solving the decision-making problem with strong sustainability [9]. In this paper, the criteria for evaluating the urban environmental sustainability is partially-compensatory. Therefore, we have a strong sustainability and weak sustainability. And the fuzzy best and worst method which improved in AHP are suitable to address this problem [9].

Only a few scholars have examined intelligent cities and environmental sustainability by applying MADM models. Anand et al. [13] evaluated the sustainability of India’s cities based on a fuzzy analytic hierarchy process (AHP) and data envelopment analysis. Wang et al. [14] assessed the impact of air pollution on urban sustainability by combining an artificial neutral network (ANN) with “the technique for order preference by similarity to an ideal solution” (TOPSIS). However, conventional methods such as AHP and the analytic network process (ANP) are difficult to implement when a considerable number of indicators are being examined. It is also difficult to obtain the accurate judgements of decision makers in the process of evaluation because their ideas are in the form of linguistic terms and are thus vague and subjective [15]. In addition, from the algorithm of these two methods, they have the problem of rank reversal. The rank reversal problem assumes that there is an alternative ranking determined for a decision problem with the use of the preferences aggregation method. For example, a right eigenvector method is used in the computational algorithm of AHP method, a left eigenvector method should use to solve a reversal problem. As a result, a reverse sequence of elements was produced to be pairwise-compared in matrix. However, this is not always true, in particular in the case of some inconsistencies in the pairwise comparison matrix [16]. Additionally, we have differences in the weights of criteria obtained by means of the ANP method with the considered cluster of alternatives or without it. Namely, taking into consideration the cluster of alternatives in the decision model influences the result of criteria weights. The effect of weight changes takes also place in the case of criteria which are not mutually dependent on each other [17].

To address these limitations, we applied a fuzzy best and worst model to obtain weights for the dimensions and criteria. The fuzzy set used in the proposed method can solve the problems related to the ambiguity and uncertainty of experts’ opinions [18]. The best worst method (BWM) developed by Rezaei has been applied in many fields because of its fast comparison times and consistent results [19,20,21,22]. The fuzzy BWM (FBWM), combining fuzzy theory with BWM, can reduce the subjective uncertainty of experts, as well as improve the accuracy of the results [23,24]. Grey relational analysis (GRA) can measure the degree of correlation between factors according to their difference or similarity. It requires a small sample size and little computation, improves the accuracy of the results, and can be used to solve MADM problems [25]. Therefore, this study also used the GRA method to assess sustainable urban environmental performance.

The objectives of this study are as follows: (i) exploring an evaluation structure for urban sustainable environmental quality, (ii) investigating the importance of environmental quality indicators for urban sustainability, and (iii) providing suggestions for improving the performance of an urban sustainable environment. The rest of the paper is organized as follows. Section 2 presents a review of the relevant literature. Section 3 describes the FBWM and GRA method. Section 4 discusses the questionnaire that was designed and implemented, as well as the analysis of the data. Section 5 discusses the results, and concluding remarks are presented in Section 6.

## 2. Literature Review

Few researchers have discussed smart or sustainable environments, and few well-recognized sustainable urban environmental evaluation systems have been developed at the national level [26,27,28]. Cicirelli et al. [29] argue that a sustainable environment is one capable of sensing, driving, communicating, and computing. The aim is to acquire and utilize environmental knowledge to meet the preferences and requirements of residents. Ahvenniemi et al. [10] proposed five dimensions to reflect sustainable environmental quality: natural environments, built environments, transportation, water management, and waste management. Aldairi et al. [11] proposed energy management, water management, waste management, clean environment, and pollution control as the five factors of a sustainable environmental assessment system. These dimensions are frequently cited and supplemented in studies of sustainable urban environments. However, the dimensions or indicators should reflect the actual environment in different countries.

Unfortunately, research on the sustainable urban environment in China is rare. Many studies have focused on analyzing the overall index of sustainable cities, but few focuses on the importance of the environmental indicators of sustainable cities. Based on a review of the literature and discussions with researchers, urban planners, and environmental experts, this study establishes a sustainable urban environmental evaluation system based on the FBWM to address this research gap in China. The sustainable urban-environmental quality-evaluation system constructed in this study comprises six dimensions and 29 criteria, as detailed in the following sections.

### 2.1. Natural Environment (D_1_)

The natural environment refers to the environment formed by natural things, such as soil, water, and climate. The urban natural environment is the foundation of urban economic and cultural development, and undoubtedly represents a crucial indicator of urban environmental sustainability. Ahvenniemi et al. [10] defined the natural environment as one element of a sustainable environmental assessment framework. Giffinge et al. [30] indicated that the attractiveness of the natural environment is a significant factor for assessing sustainable environmental quality.

The natural environment dimension comprises five major indicators: air quality, wetland area, green coverage, biodiversity, and land use. Schirnding [31] proposed an organizational framework for health, environment, and development, with air quality as a major indicator within this framework. Qian [32] emphasized that wetlands are part of the natural ecosystem and should be increased to protect the natural ecosystem. Strzelecka et al. [33] found that when assessing the quality of European smart environments, low green coverage would result in an obvious urban heat-island effect. Nader et al. [34] discussed establishing a monitoring network for environmentally sustainable development by integrating the views of government ministries, universities, research centers, and social organizations. They divided the index system into four categories: population and social-economic, economic activities, environment and sustainable development activities and policies. The green coverage rate and biodiversity were important indicators under the category of environment.

Rudden et al. [35] noted that the “European Green Capital Awards” provide a platform for cities to showcase their environmental features, and biodiversity is one of the basic criteria for this award. Land use in the territorial adjustment index system is used to measure the environmental statuses of nature reserves [36]. Serbanica and Constantin [37] observed that sustainable innovation and intelligent specialization in energy efficiency, effective land use, and waste management contributed to the sustainable development of Eastern European cities. In summary, these indicators reflect the extent to which factor in the natural environment dimension affect the quality of sustainable urban environments.

### 2.2. Artificial Environment (D_2_)

The artificial environment refers to the environment built by people in the process of natural transformation and represents a common dimension when analyzing sustainable urban environmental quality. Ahvenniemi et al. [10] observed that the artificial environment is an essential criterion for assessing the sustainability and livability of an urban environment because of its prominent role in urban environmental pollution. This study regards the artificial environment as a dimension of the sustainable urban environmental quality evaluation system.

The artificial environment comprises five major evaluation indicators: green belt area, public health, sustainable transportation, green infrastructure, and green building. Rudden et al. [35] highlighted the beautifying effect of green belts on the urban environment through the example of Vitoria, which won the United Nations Habitat Best Practice Award for improving its living environment by restoring wetlands and increasing green belt areas. Kylili [38] measured the sustainability of an artificial environment using the key performance indicator (KPI) method and emphasized public health as a major environmental performance indicator. Farizkha et al. [39] observed that green and low-carbon urban infrastructure and sustainable public transport are indicators of artificial environmental dimensions. Yi et al. [40] proposed zero-energy green buildings as an ideal choice for achieving sustainable environmental development, ensuring the symbiotic development of buildings and other environmental systems and enhancing the comfort of human settlements.

### 2.3. Energy Management (D_3_)

Energy management mainly refers to management of the processes of energy production and consumption. Energy management is used frequently when analyzing sustainable urban environmental quality. Causone et al. [41] argued that reducing energy use and improving energy efficiency should be major aspects of smart city planning. Alencar et al. [12] regarded energy management as the pillar of sustainable urban environmental development. Wang et al. [14] noted the great benefits of improving energy management for an intelligent urban environment. Therefore, this paper employs energy management as a dimension of the intelligent urban environmental quality evaluation system.

Energy consumption, clean energy, heat energy management, and energy efficiency are the four indicators in the energy management dimension. Global energy consumption represents approximately 8920 trillion tons of oil per year and may rise to 14,000 trillion tons per year by 2020 [42]. Reducing energy consumption is the first principle of sustainable development [43]. Kwatra et al. [44] observed that effective and clean energy use is an essential feature of sustainable society and a major indicator of regional sustainability. Kylili [38] identified heat energy management as the key performance index of economic sustainability. Allouhi et al. [42] argued that energy efficiency will become a global energy challenge in the 21st Century, and that various policies and measures should be adopted to improve it.

### 2.4. Water Management (D_4_)

The quality of water resource use and treatment is among the major criteria for measuring environmental sustainability. Water resources represent the core of the sustainable development of smart cities [44]. Alencar et al. [12] regarded water management as one of the three basic objectives for achieving sustainable environmental development, and the storage and reuse of rainwater are essential parts of this objective. Wastewater treatment is a vital dimension of the sustainable water resource development blueprint in smart cities [33]. Kylili [38] observed that recycling water is a key performance indicator of water management. Schirnding [31] suggested that water quality can reflect a city’s health and environmental conditions. Therefore, wastewater treatment, water quality, reuse of reclaimed water, and rainwater use are the four criteria under the water management dimension.

### 2.5. Waste Management (D_5_)

Waste management primarily refers to the management of solid waste. Waste management has become an indispensable dimension in assessing intelligent urban environmental quality because of its major role in maintaining a city’s image and environmental health [28,33,45,46]. Liu et al. [47] observed that with population growth and ongoing urbanization, waste management has become a key global problem, and waste disposal facility selection is closely related to the environment.

Alencar et al. [12] argued that improving waste management quality is a basic goal for achieving sustainable environmental development and that the production and treatment of recoverable materials is the primary guarantee of waste management. Eriksson et al. [48] emphasized that food waste represents a large proportion of organic waste and that food waste management should be strengthened. Moreover, food waste is likely to rot during collection and transportation, thus producing harmful compounds that can cause environmental hazards [49]. Mapar et al. [50] ranked hazardous waste management among the 80 sustainable development performance indicators for megacities. Kılkış [28] evaluated the environmental sustainability performance of 12 cities in Southeastern Europe and found that urban garbage management was one of the most important assessment criteria. Based on this analysis, recoverable material treatment, food waste management, hazardous waste management, and the management of other waste are the four waste management indicators in this study.

### 2.6. Pollution Control (D_6_)

Pollution control refers to the adoption of technical, economic, legal, and other means and methods for eliminating and reducing environmental pollution. It is an essential aspect that cannot be neglected in any study of sustainable urban environment. Nader et al. [34] established a monitoring network for environmentally sustainable development and created an index system that is divided into four major categories. Destruction of the ozone layer and acoustical environmental quality were the major indicators under the environmental category. Girardi and Temporelli [51] evaluated the environmental sustainability of smart cities through qualitative and quantitative indicators, such as greenhouse gas emissions and SO_2_ concentrations. Kılkış [38] proposed the annual mean PM_10_ concentrations as an index for the environmental quality dimension. In a ranking of urban sustainability assessment, 106 cities were evaluated using 46 basic indicators such as SO_2_ concentration and annual average PM_10_ concentration [52]. Cicirelli et al. [29] used sensors to measure brightness and noise levels and evaluate the climate comfort of a smart city. Cook et al. [46] established a performance index system to measure environmental sustainability and employed the carbon intensity of economic activity as an indicator of air quality and pollution.

Based on this analysis, the seven criteria under pollution control used in this study were greenhouse gas emissions, annual mean PM_10_ concentration, SO_2_ concentration, ozone layer destruction, acoustical environmental quality, brightness level, and carbon strength. Table 1 lists each criterion and the associated literature for all selected evaluation structures. Four experts were asked to verify the consistency and redundancy of this evaluating framework, including representatives from academia, government, environmental protection associations, and the environmental movement.

## 3. Methodology

**Figure 1:**
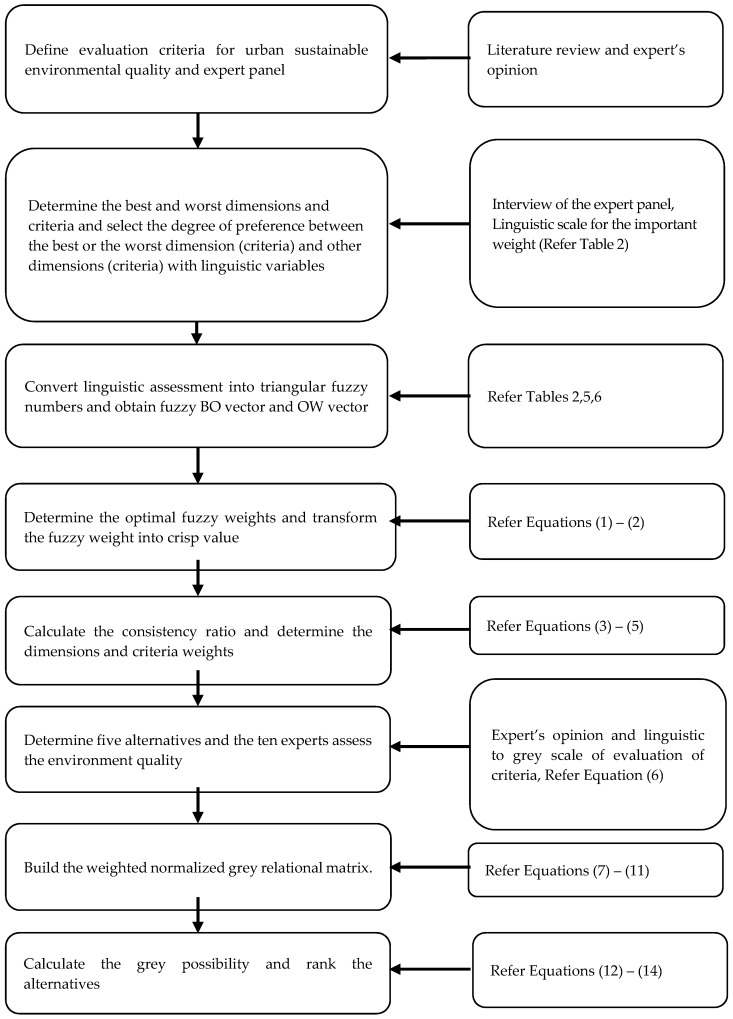
Research framework.

In order to evaluate a sustainable urban environment, we propose a MADM framework based on FBWM and GRA. As shown in Figure 1, the framework consists of three key phases: screening criteria (Section 2), obtaining optimal weights (Section 3.1) and evaluating city performance (Section 3.2). The details are as follows.

### 3.1. Fuzzy Best and Worst Method

The basic MADM methods for calculating weights are analytic hierarchy process (AHP), analytic network process (ANP), decision-making trial and evaluation laboratory-based ANP, and hybrid methods such as fuzzy AHP and fuzzy ANP. However, when an evaluation system has a very large number of indicators, the number of paired comparisons between indicators will be similarly excessive, thus rendering weight calculation extremely difficult. As a relatively new MADM method, BWM can obtain criterion weights more easily and accurately with less comparison time and higher consistency [19]. Guo and Zhao [24] proposed a hybrid model that combines fuzzy methods with BWM to improve decision accuracy. Mou et al. [23] proposed an intuitionist fuzzy multiplicative BWM for group decision-making. Hafezalkotob and Hafezalkotob [18] suggested a new method that combines individual and group decisions based on FBWM.

In this study, we examined the fuzzy preference degrees of all criteria in the form of triangular fuzzy sets. Triangular fuzzy set theory was developed to solve fuzzy and uncertain problems and can improve data accuracy based on fuzzy mathematics. A triangular-shape membership function is easy to understand and can convert uncertain data into a lower bound, middle bound and upper bound, which is more consistent with the semantics of human thought expression. Linguistic variables such as “equally important (EI),” “slightly important (SI),” “fairly important (FI),” “very important (VI),” and “absolutely important (AI)” are used to reflect the degree of preference between the best or worst criteria and other criteria. Therefore, the linguistic variables must be transformed into triangular fuzzy numbers (TFNs), with the rules of transformation listed in Table 2 [53].

We next built a fuzzy mathematical programming model to obtain the weights of dimensions and criteria, as follows:

Step 1: Set up a decision standard system.

In this step, the evaluation system criteria should be determined through a literature review and by obtaining expert opinions. Suppose there are *n* criteria $\left\{s_{1},s_{2},\cdots ,s_{n}\right\}$ for a research object.

Step 2: Determine the best (most important) dimension or criterion and the worst one (the least important).

In this step, the decision-maker determines the best and worst criteria based on the decision system.

Step 3: Derive the best-to-others (BO) vectors.

Determine the fuzzy preferences of the best criteria to all the others using TFNs, as listed in Table 2. The BO vectors can be described as ${\tilde{Q}}_{b}=\left({\tilde{q}}_{b1},{\tilde{q}}_{b2},\cdots ,{\tilde{q}}_{bn}\right)$, where *b* is the index of the best criterion, and ${\tilde{q}}_{bi}$ is a TNF indicating the degree of importance of the best criterion *C_b_* over criterion *C_i_*. Clearly, ${\tilde{q}}_{bb}$ = (1,1,1).

Step 4: Derive the others-to-worst (OW) vectors.

Following the same procedure as in step 3, the decision-maker determines the fuzzy preferences of all other criteria to the worst criterion using the TFNs listed in Table 2. The OW vectors can be described as ${\tilde{Q}}_{w}=\left({\tilde{q}}_{1w},{\tilde{q}}_{2w},\cdots ,{\tilde{q}}_{nw}\right)$, where *w* is the index of the worst criterion, and ${\tilde{q}}_{iw}$ is a TFN indicating the importance degree of another criterion *C_i_* over the worst criterion *Cw*. It is clear that ${\tilde{q}}_{ww}$ = (1,1,1).

Step 5: Determine the optimal fuzzy weights $\left({\tilde{w}}_{1}^{*},{\tilde{w}}_{2}^{*},\cdots ,{\tilde{w}}_{n}^{*}\right)$.

The ideal fuzzy weight value of each criterion satisfies the following equations: ${\tilde{w}}_{b}$/${\tilde{w}}_{i}$ = ${\tilde{q}}_{bi}$ and ${\tilde{w}}_{i}$/${\tilde{w}}_{w}$ = ${\tilde{q}}_{bi}$. We can obtain the dimension and criterion weights by minimizing the maximum absolute differences $\left|\frac{{\tilde{w}}_{b}}{{\tilde{w}}_{i}}-{\tilde{q}}_{bi}\right|$ and $\left|\frac{{\tilde{w}}_{i}}{{\tilde{w}}_{W}}-{\tilde{q}}_{iW}\right|$, where ${\tilde{w}}_{b}$, ${\tilde{w}}_{i}$, and ${\tilde{w}}_{W}$ are TFNs and ${\tilde{w}}_{i}$= ($l_{i}^{w},m_{i}^{w},u_{i}^{w}$), $l_{i}^{w}$ is the lower bound of the weight value of dimension or criterion *i*, $m_{i}^{w}$ is the middle bound, and $u_{i}^{w}$ is the upper bound.

Then, the optimal weight can be obtained by solving the following nonlinear constrained optimization problem [23].

min$\zeta^{*}$

(1) $$s.t.\left\{ \begin{array}{l} \left|\frac{{\tilde{w}}_{b}}{{\tilde{w}}_{i}}-{\tilde{q}}_{bi}\right|\leq \zeta^{*}\\ \left|\frac{{\tilde{w}}_{i}}{{\tilde{w}}_{W}}-{\tilde{q}}_{iW}\right|\leq \zeta^{*}\\ \sum_{i=1}^{n}R({\tilde{w}}_{i})=1\\ l_{i}^{w}\leq m_{i}^{w}\leq u_{i}^{w}\\ l_{i}^{w}\geq 0\\ i=1,2,\cdots ,n \end{array}\right.$$

where $\zeta^{*}=\left(h^{*},h^{*},h^{*}\right)$, and $R\left({\tilde{w}}_{i}\right)=\frac{l_{i}+4m_{i}+u_{i}}{6}$. This equation can be transformed to have greater detail into Equation (2):

(2) $$s.t.\left\{ \begin{array}{l} \left|\frac{\left(l_{b}^{w},m_{b}^{w},u_{b}^{w}\right)}{\left(l_{i}^{w},m_{i}^{w},u_{i}^{w}\right)}-\left(l_{bi},m_{bi},u_{bi}\right)\right|\leq \left(h^{*},h^{*},h^{*}\right)\\ \left|\frac{\left(l_{i}^{w},m_{i}^{w},u_{i}^{w}\right)}{\left(l_{W}^{w},m_{W}^{w},u_{W}^{w}\right)}-\left(l_{iW},m_{iW},u_{iW}\right)\right|\leq \left(h^{*},h^{*},h^{*}\right)\\ \sum_{i=1}^{n}R({\tilde{w}}_{i})=1\\ l_{i}^{w}\leq m_{i}^{w}\leq u_{i}^{w}\\ l_{i}^{w}\geq 0\\ i=1,2,\cdots ,n \end{array}\right.$$

where ${\tilde{q}}_{bi}$ = $\left(l_{bi},m_{bi},u_{bi}\right)$, and ${\tilde{q}}_{iw}$ = $\left(l_{iW},m_{iW},u_{iW}\right)$.

We transform the fuzzy criterion weight represented by TFN ${\tilde{w}}_{i}$ = ($l_{i}^{w},m_{i}^{w},u_{i}^{w}$) into a crisp value. The function $R\left({\tilde{w}}_{i}\right)$ is used to resolve ambiguous numbers, so that the weight of each dimension and criterion can be obtained.

Step 6: Determine the consistency ratio (CR) for BWM.

CR is a crucial indicator for determining the consistency of pairwise comparisons. A comparison is fully consistent when ${\tilde{q}}_{bi}\times {\tilde{q}}_{iw}={\tilde{q}}_{bw}$, where ${\tilde{q}}_{bi}$, ${\tilde{q}}_{iw}$, and ${\tilde{q}}_{bw}$ are the fuzzy preference of the best criterion over criterion *i*, the fuzzy preference of criterion *i* over the worst criterion, and the fuzzy preference of the best criterion over the worst criterion, respectively. CR can indicate the degree of consistency of a fuzzy pairwise comparison.

Guo and Zhao [24] proposed a method for calculating CR. Given that inconsistency in a fuzzy pairwise comparison occurs when ${\tilde{q}}_{bi}\times {\tilde{q}}_{iw}\neq {\tilde{q}}_{bw}$, the maximum inconsistency occurs when ${\tilde{q}}_{bi}={\tilde{q}}_{iw}={\tilde{q}}_{bw}$, and the variable ζ can be obtained to satisfy Equation (3).

(3) $$\left({\tilde{q}}_{bw}-\zeta \right)\times \left({\tilde{q}}_{bW}-\zeta \right)=\left({\tilde{q}}_{bW}+\zeta \right)$$

Guo and Zhao [18] considered that the upper boundary $u_{bw}$ could be used to calculate the CR, and thus Equation (3) can be transformed into Equation (4):

(4) $$\zeta^{2}-\left(1+2u_{bW}\right)\zeta +\left(u_{bW}^{2}-u_{bW}\right)=0$$

where ${\tilde{q}}_{bw}=\left(l_{bw},m_{bw},u_{bw}\right)$.

According to Table 2, the values of $u_{bW}$ are as follows: $u_{bW}$ = 1,3,4,5,6,7,8,9,10. The maximum possible ζ, which is considered to be consistency index (CI), can be derived using Equation (4). The CIs for different $u_{bW}$ values are listed in Table 3.

Step 7: Determine the dimension or criterion weights.

Suppose that there are *k* experts. The weight of dimension or criterion *j* can be identified by vector $\tilde{w_{j}}=\left\{w_{j}^{1},w_{j}^{2},w_{j}^{3},\ldots ,w_{j}^{k}\right\}$, and the dimension or criterion weight can be obtained by averaging the elements $\tilde{w_{j}}$:

(5) $$w=\frac{1}{k}\left[w_{j}^{1}+w_{j}^{2}+w_{j}^{3}+\ldots +w_{j}^{k}\right]$$

### 3.2. Grey Relational Analysis

Grey relational analysis (GRA) is used to measure the degree of correlation between similarities or differences of criteria variability. One advantage of GRA is that it can provide a high-quality result with a small sample size and little calculation. It can be applied to decision-making in multiple-attribute decision problems [25,53]. Therefore, GRA was applied to the performance evaluation of sustainable urban environmental quality. Rajesh and Ravi [54] proposed a GRA method with an interval probability algorithm, that has the following steps:

Step 1: Determine the number of alternatives.

The suitable *m* alternatives are chosen based on decisions in the sustainable urban environment quality assessment. Let $X=\left\{X_{1},X_{2},X_{3},\ldots ,X_{m}\right\}$ be *m* sets of alternatives.

Step 2: Linguistic to grey scale of evaluation of criteria.

Expert *h* assessing the performance of criterion *j* of alternative *i* can be represented as $V_{ij}^{h}$, where i={1,2,3,4…,m}, j={1,2,3,4…,n}, and h={1,2,3,4,…,k}. The linguistic variable $V_{ij}^{h}=\left\lfloor {\underline{V}}_{ij}^{h},{\bar{V}}_{ij}^{h}\right\rfloor$ can be obtained using the grey relational semantic transformation rule, where ${\underline{V}}_{ij}^{h}$ is the lower bound of the grey value $V_{ij}^{h}$, and ${\bar{V}}_{ij}^{h}$ is the upper bound.

The average value can be calculated as follows:

(6) $$⊗V_{ij}=\left[\left(\frac{1}{k}\sum_{h=1}^{k}({\underline{V}}_{ij}^{h})\right),\left(\frac{1}{k}\sum_{h=1}^{k}({\bar{V}}_{ij}^{h})\right)\right]$$

Step 3: Build the grey matrix.

Grey matrix *M* is obtained from the average grey values $⊗V_{ij}$.

(7) $$M=\left[\begin{array}{cccc} ⊗V_{11} & ⊗V_{12} & \cdots & ⊗V_{1n}\\ ⊗V_{21} & ⊗V_{22} & \cdots & ⊗V_{2n}\\ \vdots & \vdots & \ddots & \vdots \\ ⊗V_{m1} & ⊗V_{m2} & \cdots & ⊗V_{mn} \end{array}\right]$$

Step 4: Normalize the grey relational matrix.

The grey number between limits [0,1] can be obtained through normalization, as follows:

(8) $$⊗V=\left[\frac{{\underline{V}}_{ij}}{V_{j}^{\max}},\frac{V_{ij}}{V_{j}^{\max}}\right]$$

where $V_{j}^{\max}{=}_{1\leq i\leq m}^{\max}\left\{{\bar{V}}_{ij}\right\}$.

The normalized grey relational matrix M* is represented as follows:

(9) $$M^{*}=\left[\begin{array}{cccc} ⊗V_{11}^{*} & ⊗V_{12}^{*} & \cdots & ⊗V_{1n}^{*}\\ ⊗V_{21}^{*} & ⊗V_{22}^{*} & \cdots & ⊗V_{2n}^{*}\\ \vdots & \vdots & \ddots & \vdots \\ ⊗V_{m1}^{*} & ⊗V_{m2}^{*} & \cdots & ⊗V_{mn}^{*} \end{array}\right]$$

Step 5: Build the weighted normalized grey relational matrix.

The weighted normalized matrix ($⊗E_{ij}$) can be obtained when the weight ($w_{j}$) calculated using FBWM is multiplied by the normalized grey relational matrix ($⊗V_{ij}^{*}$):

(10) $$⊗E_{ij}=\left[\left(⊗V_{ij}^{*}\right)*w_{j}\right]$$

where $⊗V_{ij}^{*}=\left\lfloor {\underline{V}}_{ij}^{h},{\bar{V}}_{ij}^{h}\right\rfloor$

The weighted normalized grey relational matrix M** is represented as follows:

(11) $$M^{**}=\left[\begin{array}{cccc} ⊗E_{11} & ⊗E_{12} & \cdots & ⊗E_{1n}\\ ⊗E_{21} & ⊗E_{22} & \cdots & ⊗E_{2n}\\ \vdots & \vdots & \ddots & \vdots \\ ⊗E_{m1} & ⊗E_{m2} & \cdots & ⊗E_{mn} \end{array}\right]$$

Step 6: Build the ideal referential set of alternatives.

We can obtain the maximum $⊗E_{ij}$ of all columns by comparing the values of each column in matrix $M^{**}$. The maximum $⊗E_{ij}$ is denoted as $X^{\max}$:

(12) $$X^{\max}=\left[\begin{array}{c} \left[\overset{\max}{1}\leq i\leq m{\underline{E}}_{i1},\overset{\max}{1}\leq i\leq m{\bar{E}}_{i1}\right],\\ \left[\overset{\max}{1}\leq i\leq m{\underline{E}}_{i2},\overset{\max}{1}\leq i\leq m{\bar{E}}_{i2}\right],\\ \left[\overset{\max}{1}\leq i\leq m{\underline{E}}_{i3},\overset{\max}{1}\leq i\leq m{\bar{E}}_{i3}\right],\\ \left[\overset{\max}{1}\leq i\leq m{\underline{E}}_{in},\overset{\max}{1}\leq i\leq m{\bar{E}}_{in}\right] \end{array}\right]=\left\{C_{1}^{\max},C_{2}^{\max},C_{3}^{\max},\ldots ,C_{n}^{\max}\right\},$$

where $C_{i}^{\max}$ = $\left\lfloor {\underline{C}}_{i}^{\max},{\bar{C}}_{i}^{\max}\right\rfloor$.

Step 7: Calculate the grey possibility by comparing *X_i_* with $X^{\max}$.

By comparing the alternatives set *X_i_* with the ideal referential $X^{\max}$, we can obtain the grey possibility for each alternative, which is given as follows:

(13) $$p(X_{i}\leq X^{\max})=\frac{1}{n}\sum_{j=1}^{n}\left[\frac{\max\left(0,L_{j}^{*}-\max\left(0,{\bar{E}}_{ij}-{\underline{C}}_{j}^{\max}\right)\right)}{L_{j}^{*}}\right]$$

where $L_{j}^{*}$ is the sum of length $⊗E_{ij}$ and $C_{j}^{\max}$, which can be represented as follows:

(14) $$L_{j}^{*}=L(⊗E_{ij})+(⊗C_{j}^{\max})$$

Equation (13) can be transformed as follows:

(15) $$L_{j}^{*}=\left\lfloor \left({\bar{E}}_{ij}-{\underline{E}}_{ij})+({\bar{C}}_{j}^{\max}-{\underline{C}}_{j}^{\max}\right)\right\rfloor$$

Step 8: Rank the alternatives.

We can sort the alternatives after obtaining the probability value $p(X_{i}\leq X^{\max})$. An alternative is closer to the ideal referential when its possibility value is lower.

## 4. Results Analysis

In this section, we apply the proposed hybrid model combining FBWM with GRA to evaluate the sustainable urban environmental quality of five Chinese cities: Guangzhou, Shanghai, Beijing, Hangzhou, and Shenzhen. These five cities are in highly developed regions of China and play a vital role in its economy and culture. Beijing is the capital of China. It is a cultural, political and educational center. Shanghai is China’s most economically developed city and the most populous city. It is China’s financial and technological development center. Guangzhou is the largest city in South China with developed manufacturing and commerce. Shenzhen is China’s most dynamic entrepreneurial city. Its GDP rate is growing fast, and its total GDP has surpassed that of Guangzhou, ranking third. Hangzhou is the representative of the new first-tier cities in China. It is China’s e-commerce capital, there are lots of famous internet companies, such as Alibaba and NetEase. These five cities have been devoted to environmental improvement and smart city construction for a long time. For other cities, based on the familiarity with the advantages and disadvantages of the five cities in a sustainable environment, managers or government can serve as a reference to improve their own urban environment for future development.

To perform a comprehensive evaluation, we selected 10 Chinese experts with abundant experience in different fields. The group of experts comprised three professors from the Institute of Environmental Engineering, two managers at an environmental monitoring company, one researcher from the Intelligent City Institute, one researcher from the Intelligent Environment Institute, one manager at a pollution testing company, and two officials from the Environmental Protection Bureau. The duties of these 10 experts are closely related to the urban environment, including urban environment governance, urban environmental engineering design, environmental pollution detection, and urban environment research. These experts have more than 10 years of work experience. Although they come from different work backgrounds, their different evaluation perspectives are deemed as having equal importance. Experts from enterprises and research institutes are members of the National Urban Environmental Planning Expert Pool. They are very familiar with the urban environment of major cities in China. Government personnel are engaged in urban environmental management and often understand the environmental development of various cities in China. The experts were asked to answer a two-part questionnaire. The first part of the questionnaire was used to assess the importance of the six dimensions and 29 criteria, and the second part was used to rate the performance of the five cities with respect to the 29 criteria. It took three months from November 2017 to January 2018 to contact 10 experts to fill out the questionnaire.

### 4.1. Determination of Criteria Weights

The analytical processes consisted of the seven steps of fuzzy best and worst method introduced in Section 3.1. They were used to obtain the weights of the dimensions and criteria and as a basis for performance evaluation. The experts were asked to identify the most important of the six dimensions in Table 1 and the most important criterion within each dimension. Similarly, the least important dimension and criteria were decided based on the experts’ opinions. Table 4 displays the best and worst dimensions identified by the experts. One expert stated that natural environment (*D*_1_) was the most important dimensions, and another argued that energy management (*D*_3_) was the best. The eight other experts all selected pollution control (*D*_6_) as the best dimension. All experts unanimously deemed artificial environment (*D*_2_) as the worst of the six dimensions. The best and worst criteria within each dimension were obtained in the same manner.

After selecting the best and worst dimensions and criteria, the experts were asked to determine the preference of the best ones over all others and the preferences of all others over the worst dimension or criterion using the linguistic variables proposed in Section 3. As shown in Table 5, the third expert considered pollution control (*D*_6_) to be more important (between FI and VI) than artificial environment (*D*_2_) with the interval value (5,6,7) obtained according to Table 2. As shown in Table 6, the first expert believed that natural environment (*D*_1_) was slightly more important than the worst dimension (artificial environment, *D*_2_), with the interval value (2,3,4). The preference values of the best criterion over all other criteria within a dimension and of all others over the worst, were obtained through the same procedure.

The weights of the dimensions and criteria were calculated using a linear model for the experts according to Equation (2). Since the experts come from different departments and have different job responsibilities, their assessments reflect different perspectives. All of the experts have many years of work experience related to the urban environment, and the importance of each expert’s opinion is considered equal [20,55]. The average weight for each dimension and criterion for the experts was obtained, which are ranked by value in Table 7.

We calculated the CRs of the dimensions for the experts, and all were below 0.1. A smaller value indicates a higher consistency in pairwise comparisons.

The results showed that pollution control (*D*_6_, 29.3%) accounted for the highest weight in the evaluation system, and ozone layer destruction (*C*_64_, 6.4%) ranked fifth among the 29 criteria. Therefore, more attention should be given to ozone layer damage by strengthening the monitoring of harmful substances and incorporating such monitoring in the air evaluation system of the intelligent city environment. The government should also advocate the use of environmentally friendly appliances such as Freon-free refrigerators.

Energy consumption (*C*_31_, 0.07%) ranked fourth and hazardous waste management (*C*_53_, 0.075%) ranked first among the 29 criteria. This indicates that enterprises should improve their production processes, develop advanced industrial manufacturing, reduce the direct discharge of waste heat, and implement waste heat recycling Urban waste treatment cannot be ignored. It is necessary to popularize garbage sorting activities, promote urban coverage of garbage sorting facilities, promote rational and efficient food waste use, promote clean cities, and create good conditions through different forms of communication, such as the government’s public WeChat channel, publicity handbooks, and banners.

### 4.2. Sustainable Urban Environmental Quality Evaluation

After obtaining the weights of dimensions and criteria, we evaluated the sustainable environmental quality of each city using the GRA method outlined in Section 3.2. We chose Guangzhou (*X*_1_), Shanghai (*X*_2_), Beijing (*X*_3_), Hangzhou (*X*_4_), and Shenzhen (*X*_5_) as the five cities for this cases study because they are in China’s top five developed regions.

The experts evaluated the sustainable environmental quality of the cities based on their knowledge and experience. We applied grey preference degrees to all criteria to evaluate the performance of the cities in the form of grey intervals. Linguistic variables were used to rate performance of the alternatives (cities) for the corresponding criterion: “very poor (VP)”, “poor (P)”, “medium poor (MP)”, “fair (F)”, “medium good (MG)”, “good (G)”, and “very good (VG)”. $V_{ij}^{h}$ denotes the value that expert *h* assigned to criterion *j* for city *i* and can be represented using the grey number $V_{ij}^{h}=\left\lfloor {\underline{V}}_{ij}^{h},{\bar{V}}_{ij}^{h}\right\rfloor$ according to the rules in Table 8 [48].

The average evaluation value $⊗V_{ij}$ of criterion *j* for city *i* among the experts was calculated using Equation (6), and the grey decision matrix *M* was obtained using Equation (7), as shown in Table A1 of Appendix A. The normalized grey decision matrix M* obtained using Equation (8) is shown in Table A2 of Appendix A. The weighted normalized grey decision matrix M** was obtained using Equations (10) and (11), as illustrated in Table A3 of Appendix A. The ideal referential set of alternatives $X^{\max}$ can be obtained using Equation (12). The overall grey possibility $p(X_{i}\leq X^{\max})$ can be calculated using Equations (13)–(15), and the grey possibility of sustainable environmental quality for the five cities is as follows: $P(X_{1}<X^{\max})$ = 0.91194, $P(X_{2}<X^{\max})$ = 0.95519, $P(X_{3}<X^{\max})$ = 0.85828, $P(X_{4}<X^{\max})$ = 0.64435, and $P(X_{5}<X^{\max})$ = 0.73386 (see Table 9).

The smaller the probability of the grey relation is the closer to the ideal alternative [54]. Therefore, the cities can be ranked according to sustainable environmental quality as follows: $P(X_{4}<X^{\max})$ > $P(X_{5}<X^{\max})$ > $P(X_{3}<X^{\max})$ > $P(X_{1}<X^{\max})$ > $P(X_{2}<X^{\max})$. The best sustainable environmental quality was thus found in Hangzhou, followed by Shenzhen, Beijing, and Guangzhou, with the worst sustainable environmental quality performance noted in Shanghai.

## 5. Discussion

Table A1 in Appendix A shows that the scores for green infrastructure (*C*_24_), wetland area (*C*_12_), green coverage rate (*C*_13_), and air quality (*C*_11_) were highest, and the maximum upper boundaries of the scores were given by the experts as $⊗V_{24}=9.4,$ $⊗V_{12}=9.3,$ $⊗V_{13}=9.3$, and $⊗V_{11}=9.1$, respectively. Therefore, the sustainable environment construction in the five cities has achieved remarkable results in these four areas. There seems to be a consensus on using water-saving faucets in washrooms and installing green infrastructure such as electric panels on buildings. With the popularization of cultural and scientific knowledge in China, citizens are also paying increasing attention to green vegetation and the roles as the city’s “kidneys” and “lung”. China’s government has placed great emphasis on ecological environment construction, the protection and renewal of vegetation, and increased vegetation coverage. Moreover, cities in China are more committed to improving air quality. In Beijing, for example, air quality has been greatly improved by limiting the number of vehicles and issuing license plates.

For the importance of dimensions (Table 7), the results indicated that pollution control (*D*_6_, 0.293), natural environment (*D*_1_, 0.192), and water management (*D*_4_, 0.178) were the three most influential dimensions for urban sustainable environmental quality. This is consistent with the conclusions of other studies that air pollution and water resource quality are important indicators of urban environments [56,57,58]. Compared to other criteria, Table A1 shows that the performance values for heat energy management (*C*_33_), materials treatment (*C*_51_), and ozone layer destruction (*C*_64_) were significantly lower (the average upper boundaries among the experts were 6.3, 7.2, and 7.5, respectively). Therefore, shortcomings remain in sustainable urban environment construction in these areas. As shown in Table 7, the weight of the ozone layer destruction (*C*_64_, 0.064) accounts for 6.424% of the total weight of the urban sustainable environmental quality evaluation system, which means that it is a significant urban environmental indicator. However, many cities still ignore this aspect in weather forecasting and air monitoring and have not established an improved monitoring system, which could hinder the construction of a city environment’s sustainability.

Waste treatment (*D*_5_, 0.151) is essential for maintaining a clean urban environment. Hazardous waste management (*C*_53_, 0.075) accounts for 7.5% of the assessment system weight and thus has the highest weight among all 29 criteria (Table 7). Its smallest lower-boundary grey value is 0.62, which is larger than that of the other 28 criteria. The Chinese government has realized that hazardous waste causes serious environmental destruction and has banned waste imports as part of its campaign against “foreign garbage.” Garbage-sorting experiments have been carried out in developed cities such as Hangzhou and Xiamen, but public awareness of this practice must be further improved. Some large cities such as Guangzhou have not implemented garbage sorting and lack food waste management. Garbage sorting is beneficial for the rational and efficient use of waste resources.

The way of using waste heat (*C*_33_, 0.011) from urban economic production processes and daily life activities is not effective. This not only causes energy waste but also produces more waste heat directly in the air, thus further increasing the urban heat-island effect. Therefore, it is necessary to strengthen heat energy management in production processes, develop advanced industrial manufacturing, promote the reuse of waste-heat resources, promote clean production processes, and undertake sustainable city development.

When experts evaluate sustainable urban environmental quality, they may not consider all the indicators and instead use a single index because of differences in personal values and preferences. This can affect their overall evaluation of the sustainable city environment. Based on a single index of sustainable environmental quality for each city, a single indicator of the five cities’ environmental quality was created, as presented in Table 9. For example, if air quality is a highly preferred indicator, then the quality of the intelligent city environment in Shenzhen would be considered the best.

Mangla et al. [59] and Guota and Barua [60] suggested a sensitivity analysis method for verifying the robustness of an evaluation system and eliminating biases. Therefore, to verify the effects of weights in our proposed model, we selected the highest weight from among the 29 criteria (hazardous waste management, *C*_53_) and varied it from 0.1 to 0.9 (Table 10). All other criteria weights were found to correspondingly change with it. A resulting variety in criteria ranking was observed. The five cities were then ranked using GRA in 9 different runs, and their ranks were compared, as shown in Table 11. The sensitivity results indicated that the model proposed in this paper was robust.

**Figure 2:**
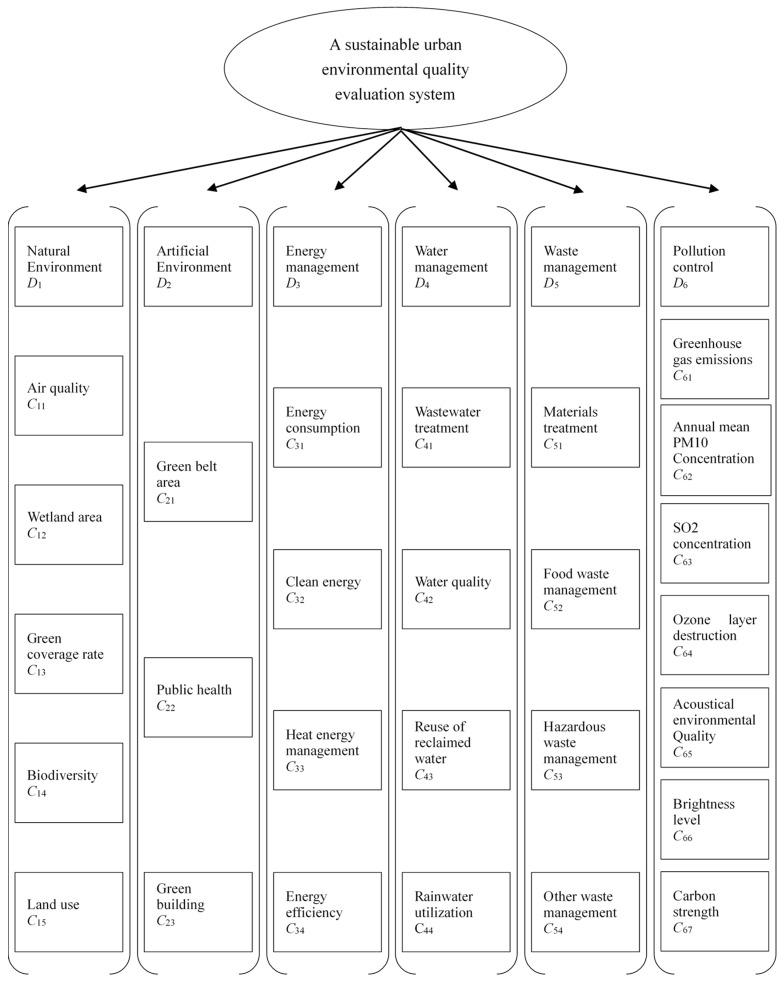
A sustainable urban environmental quality evaluation system.

We also applied another sensitivity analysis method to test whether the indicators ranked last have an impact on performance evaluation. Interestingly, we found proof that the four lowest-ranked indicators were in the artificial environment dimension. First, we deleted the lowest-ranked criterion (sustainable transportation) and found that the results of the performance evaluation remained stable. Next, we removed green infrastructure, and the results remained unaffected. However, the results of the performance evaluation showed large fluctuations when we deleted the third-lowest indicator, green building. Therefore, we conclude that sustainable transportation and green infrastructure have no significant impact on the assessment of sustainable urban environment in China. Therefore, we believe that the system for evaluating sustainable urban environment quality involves a total of 27 indicators in six dimensions, as shown in Figure 2. Local government in China are devoting their energies to air pollution and water pollution control and management in cities [61,62]. Enterprises under the guidance of the government also began to pay attention to the effective use of energy [63]. These government and enterprise actions further validate the rationality and application value of our model.

In Summary, the proposed hybrid model provides a systematic way to evaluate sustainable urban environment and suggest improvement measures. The sustainability of urban environments is an important issue in urban development and management. The proposed model has not been applied previously in sustainable urban environment evaluation. The model adopts fuzzy and grey techniques to solve the problems related to the subjectivity and information uncertainty in the assessments of experts. The practicality and effectiveness of the proposed model was also demonstrated through a sensitivity analysis. The findings could provide various advantages in terms of (i) deciding the most appropriate criteria for sustainable urban environment evaluation, (ii) applying an advanced model to find the weights of the dimensions and criteria of the evaluation system, (iii) providing a highly reliable assessment of urban sustainability environmental performance, (iv) and providing targeted measures for the improvement of the urban environmental sector based on experts’ judgements. This evaluation system could provide administrations with a guideline for sustainable city development.

## 6. Conclusions

This study proposed six dimensions and 27 criteria to evaluate urban environmental quality. The dimensions comprise the natural environment, artificial environment, energy management, water management, waste management, and pollution control. A hybrid MADM model was proposed for construction a sustainable urban-environmental quality-evaluation system. FBWM was used to calculate the weights of the criteria, and then GRA was applied with a possibility interval algorithm to obtain the sustainable environmental quality performance of five Chinese cities.

The results indicated that pollution control, natural environment, and water management were the three most influential dimensions for urban sustainable environmental quality. Therefore, controlling pollutant emissions, cleaning air and water, improving clean production processes, and reducing emissions of ozone layer materials represent the most essential tasks for the government and the public. The results of GRA with a possibility interval algorithm showed that the overall levels of sustainable environmental quality in Shenzhen and Hangzhou was good, whereas Beijing had favorable performance for water management but poor performance for natural environment and pollution control. Therefore, the public and the government should devise strategies to improve the natural environment and pollution control quality in this city.

There are some limitations in the application of the hybrid model. We interviewed experts and analyzed the data. Although the average CR was 0.73%, we cannot conclude that this represents the consensus of all stakeholders. Fuzzy and grey techniques were used to reduce the experts’ subjective bias due to their different backgrounds. Other methods could also be used to address this problem, such as Delphi or artificial intelligence methods. If real data can be collected, it could be possible to apply multiple-objectives, decision-making methods, and data mining techniques to obtain more reasonable conclusions. The empirical data were limited to five cities in China, and therefore, the applicability of the findings to other cities and countries may vary. Regarding future research, cases of sustainable urban environments could be collected for performance evaluation using VIKOR, TOPSIS, and other methods, based on the FBWM model in this study. The proposed model could also be used in similar decision-making problems in other fields.

## Figures and Tables

**Table 1:** Descriptions of dimensions and criteria.

Dimensions	Criteria	Definitions	Sources
Natural environment *D*_1_	*C*_11_ Air quality	Reflects the degree of air pollution	[10,30,31]
	*C*_12_ Wetland area	An ecosystem that is inundated by water	[32]
	*C*_13_ Green coverage rate	The ratio of the vertical projected area of vegetation to the total land area of the city	[33,34]
	*C*_14_ Biodiversity	The variety and variability of life on city	[10,35]
	*C*_15_ Land use	The management and modification of natural environment or wilderness into built environment	[30,37]
Artificial environment *D*_2_	*C*_21_ Green belt area	A protected area of green space, farmland, forests in city	[10,35]
	*C*_22_ Public health	Prevent disease, prolong life and promote human health through organized efforts	[38]
	*C*_23_ Sustainable transportation	The ability to supply the source energy indefinitely in city	[39]
	*C*_24_ Green infrastructure	A network providing the “ingredients” for solving urban and climatic challenges by building with nature	[39]
	*C*_25_ Green building	A structure and application of processes that are environmentally responsible	[40]
Energy management *D*_3_	*C*_31_ Energy consumption	The total energy used by the city	[41,42,43]
	*C*_32_ Clean energy	Energy that does not emit pollutants	[44]
	*C*_33_ Heat energy management	The transfer of energy between systems	[38]
	*C*_34_ Energy efficiency	The ratio between the useful output and input of an energy conversion process	[43]
Water management *D*_4_	*C*_41_ Wastewater treatment	A process used to convert wastewater to the water with minimum impact on the environment, or directly reused	[12,33,45]
	*C*_42_ Water quality	The chemical, physical, biological, and radiological characteristics of water	[31,45]
	*C*_43_ Reuse of reclaimed water	Reclaimed water can be used for other purposes	[12,32,38]
	*C*_44_ Rainwater utilization	Rainwater harvesting system, rainwater interception and infiltration system	[12,32,45]
Waste management *D*_5_	*C*_51_ Materials treatment	Use high-tech process materials to reduce environmental hazards	[12,35,47]
	*C*_52_ Food waste management	Reduce the pollution of food waste to urban environment	[48,49]
	*C*_53_ Hazardous waste management	The city adopts systems and technologies for managing hazardous waste	[50]
	*C*_54_ Other waste management	The way to manage other waste, like construction rubbish	[12,28,35]
Pollution control *D*_6_	*C*_61_ Greenhouse gas emissions	The atmosphere absorbs solar radiation reflected from the ground and re-emits some of the radiated gas, like CO_2_, NO_2_	[30,34]
	*C*_62_ Annual mean PM_10_ concentration	An average annual distribution density of particles with a particle size below 10 microns	[34,38,52]
	*C*_63_ SO2 concentration	The flue gas concentration cannot be satisfied when the contact method is self-heating to produce sulfuric acid	[34,52]
	*C*_64_ Ozone layer destruction	Degree of damage to the ozone layer over the city	[24,52]
	*C*_65_ Acoustical environmental quality	The impact of urban noise on residents’ lives	[34,52]
	*C*_66_ Brightness level	City night illumination	[52]
	*C*_67_ Carbon strength	CO_2_ emissions per unit of GDP	[46]

**Table 2:** Transformation rules of linguistic variables.

Linguistic Variables	Membership Function
Equally importance (EI)	(1,1,1)
Between the two	(1,2,3)
slightly important (WI)	(2,3,4)
Between the two	(3,4,5)
Fairly Important (FI)	(4,5,6)
Between the two	(5,6,7)
Very important (VI)	(6,7,8)
Between the two	(7,8,9)
Absolutely important (AI)	(8,9,10)

**Table 3:** CI for FBWM.

Linguistic Terms	${\tilde{q}}_{bW}$	CI
Equally importance (EI)	(1,1,1)	3.00
Between the two	(1,2,3)	6.00
Weakly important (WI)	(2,3,4)	7.36
Between the two	(3,4,5)	8.69
Fairly Important (FI)	(4,5,6)	10.00
Between the two	(5,6,7)	11.27
Very important (VI)	(6,7,8)	12.53
Between the two	(7,8,9)	13.77
Absolutely important (AI)	(8,9,10)	15.00

**Table 4:** Best and worst dimensions determined by the 10 experts.

Dimension	Determined as “Best” by Expert No.	Determined as “Worst” by Expert No.
*D*_1_	1	
*D*_2_		1,2,3,4,5,6,7,8,9,10
*D*_3_	6	
*D*_4_		
*D*_5_		
*D*_6_	2,3,4,5,7,8,9,10	

**Table 5:** BO dimension vectors for the 10 experts.

Expert No.	Best	*D*_1_	*D*_2_	*D*_3_	*D*_4_	*D*_5_	*D*_6_
1	*D*_1_	(1,1,1)	(2,3,4)	(1,1,1)	(1,1,1)	(1,1,1)	(1,1,1)
2	*D*_6_	(1,2,3)	(7,8,9)	(3,4,5)	(1,2,3)	(2,3,4)	(1,1,1)
3	*D*_6_	(1,2,3)	(5,6,7)	(1,2,3)	(1,2,3)	(1,2,3)	(1,1,1)
4	*D*_6_	(1,2,3)	(5,6,7)	(2,3,4)	(1,2,3)	(2,3,4)	(1,1,1)
5	*D*_6_	(1,2,3)	(7,8,9)	(2,3,4)	(2,3,4)	(1,2,3	(1,1,1)
6	*D*_3_	(1,1,1)	(4,5,6)	(1,1,1)	(1,1,1)	(1,1,1)	(1,1,1)
7	*D*_6_	(1,2,3)	(7,8,9)	(1,2,3)	(2,3,4)	(3,4,5)	(1,1,1)
8	*D*_6_	(1,1,1)	(5,6,7)	(1,2,3)	(1,1,1)	(1,2,3)	(1,1,1)
9	*D*_6_	(1,1,1)	(7,8,9)	(1,2,3)	(1,1,1)	(1,1,1)	(1,1,1)
10	*D*_6_	(1,2,3)	(8,9,10)	(2,3,4)	(1,2,3)	(2,3,4)	(1,1,1)

**Table 6:** OW dimension vectors for the 10 experts.

Expert No.	1	2	3	4	5	6	7	8	9	10
Worst	*D*_2_	*D*_2_	*D*_2_	*D*_2_	*D*_2_	*D*_2_	*D*_2_	*D*_2_	*D*_2_	*D*_2_
*D*_1_	(2,3,4)	(3,4,5)	(2,3,4)	(2,3,4)	(4,5,6)	(4,5,6)	(3,4,5)	(5,6,7)	(7,8,9)	(3,4,5)
*D*_2_	(1,1,1)	(1,1,1)	(1,1,1)	(1,1,1)	(1,1,1)	(1,1,1)	(1,1,1)	(1,1,1)	(1,1,1)	(1,1,1)
*D*_3_	(2,3,4)	(1,2,3)	(2,3,4)	(1,2,3)	(2,3,4)	(4,5,6)	(3,4,5)	(2,3,4)	(3,4,5)	(2,3,4)
*D*_4_	(2,3,4)	(3,4,5)	(2,3,4)	(2,3,4)	(2,3,4)	(4,5,6)	(2,3,4)	(5,6,7)	(7,8,9)	(3,4,5)
*D*_5_	(2,3,4)	(2,3,4)	(2,3,4)	(1,2,3)	(4,5,6)	(4,5,6)	(1,2,3)	(2,3,4)	(7,8,9)	(2,3,4)
*D*_6_	(2,3,4)	(7,8,9)	(5,6,7)	(5,6,7)	(8,9,10)	(4,5,6)	(7,8,9)	(5,6,7)	(7,8,9)	(8,9,10)

**Table 7:** Overall weights of dimensions and criteria.

Dimensions	Weights	Criteria	Local Weights	Global Weights	Ranking
Natural environment (*D*_1_)	0.192	*C*_11_	0.263	0.051	9
		*C*_12_	0.184	0.035	12
		*C*_13_	0.147	0.028	16
		*C*_14_	0.333	0.064	6
		*C*_15_	0.072	0.014	22
Artificial environment (*D*_2_)	0.046	*C*_21_	0.190	0.008	26
		*C*_22_	0.453	0.021	19
		*C*_23_	0.082	0.004	29
		*C*_24_	0.133	0.006	28
		*C*_25_	0.141	0.007	27
Energy management (*D*_3_)	0.140	*C*_31_	0.501	0.070	4
		*C*_32_	0.251	0.035	13
		*C*_33_	0.076	0.011	24
		*C*_34_	0.172	0.024	18
Water management (*D*_4_)	0.178	*C*_41_	0.307	0.055	8
		*C*_42_	0.415	0.074	2
		*C*_43_	0.203	0.036	11
		*C*_44_	0.075	0.013	23
Waste management (*D*_5_)	0.151	*C*_51_	0.094	0.014	21
		*C*_52_	0.228	0.034	14
		*C*_53_	0.494	0.075	1
		*C*_54_	0.185	0.028	17
Pollution control (*D*_6_)	0.293	*C*_61_	0.240	0.070	3
		*C*_62_	0.106	0.031	15
		*C*_63_	0.205	0.060	7
		*C*_64_	0.219	0.064	5
		*C*_65_	0.034	0.010	25
		*C*_66_	0.058	0.017	20
		*C*_67_	0.138	0.040	10

**Table 8:** Linguistic assessment and associated grey values.

Associated Grey Numbers		Linguistic Assessment
Lower Bound 0	Upper Bound 1	Rating of Attributes Very Poor (VP)
1	3	Poor (P)
3	4	Medium Poor (MP)
4	5	Fair (F)
5	6	Medium Good (MG)
6	9	Good (G)
9	10	Very Good (VG)

**Table 9:** Quality rankings of sustainable urban environments for 29 indices.

Criteria	P(X_i_ ≤ X_max_)					
	P(X_1_ ≤ X_max_)	P(X_2_ ≤ X_max_)	P(X_3_ ≤ X_max_)	P(X_4_ ≤ X_max_)	P(X_5_ ≤ X_max_)	Priority
*C*_11_	0.773	1.000	1.000	0.700	0.500	X_5_ > X_4_ > X_1_ > X_2_ = X_3_
*C*_12_	1.000	1.000	1.000	0.500	1.000	X_4_ > X_1_ = X_2_ = X_3_ = X_5_
*C*_13_	0.944	1.000	1.000	0.500	1.000	X_4_ > X_1_ > X_2_ = X_3_ = X_5_
*C*_14_	0.824	1.000	1.000	0.500	1.000	X_4_ > X_1_ > X_2_ = X_3_ = X_5_
*C*_15_	1.000	0.970	0.533	0.842	0.579	X_3_ > X_5_ > X_4_ > X_2_ > X_1_
*C*_21_	0.630	1.000	0.775	0.500	0.800	X_4_ > X_1_ > X_3_ > X_5_ > X_2_
*C*_22_	1.000	1.000	0.868	0.500	0.743	X_4_ > X_5_ > X_3_ > X_1_ = X_2_
*C*_23_	1.000	1.000	0.694	0.500	0.649	X_4_ > X_5_ > X_3_ > X_1_ = X_2_
*C*_24_	1.000	1.000	1.000	0.500	1.000	X_4_ > X_1_ = X_2_ = X_3_ = X_5_
*C*_25_	1.000	1.000	0.639	0.500	1.000	X_4_ > X_3_ > X_1_ = X_2_ = X_5_
*C*_31_	0.790	1.000	1.000	0.500	0.632	X_4_ > X_5_ > X_1_ > X_2_ = X_3_
*C*_32_	0.810	1.000	1.000	0.548	0.500	X_5_ > X_4_ > X_1_ > X_2_ = X_3_
*C*_33_	0.960	0.583	1.000	0.792	0.533	X_5_ > X_2_ > X_4_ > X_1_ > X_3_
*C*_34_	1.000	0.667	1.000	1.000	0.500	X_5_ > X_2_ > X_1_ = X_3_ = X_4_
*C*_41_	1.000	1.000	1.000	0.500	1.000	X_4_ > X_1_ = X_2_ = X_3_ = X_5_
*C*_42_	1.000	1.000	0.500	1.000	0.591	X_3_ > X_5_ > X_1_ = X_2_ = X_4_
*C*_43_	1.000	1.000	0.500	1.000	0.643	X_3_ > X_5_ > X_1_ = X_2_ = X_4_
*C*_44_	1.000	1.000	0.500	1.000	0.895	X_3_ > X_5_ > X_2_ = X_3_ = X_1_
*C*_51_	1.000	0.821	0.964	0.5000	0.969	X_4_ > X_2_ > X_3_ > X_5_ > X_1_
*C*_52_	1.000	1.000	0.711	1.000	0.500	X_5_ > X_3_ > X_1_ = X_2_ = X_4_
*C*_53_	0.6410	0.966	0.811	0.630	0.541	X_5_ > X_4_ > X_1_ > X_3_ > X_2_
*C*_54_	1.000	1.000	0.644	0.538	0.512	X_5_ > X_4_ > X_3_ > X_1_ = X_2_
*C*_61_	0.929	1.000	1.000	0.500	0.813	X_4_ > X_5_ > X_1_ > X_2_ = X_3_
*C*_62_	0.756	1.000	1.000	0.622	0.511	X_5_ > X_4_ > X_1_ > X_2_ = X_3_
*C*_63_	0.829	1.000	1.000	0.515	0.541	X_4_ > X_5_ > X_1_ > X_2_ = X_3_
*C*_64_	0.923	1.000	1.000	0.500	1.000	X_4_ > X_1_ > X_2_ = X_3_ = X_5_
*C*_65_	0.639	0.889	0.9167	0.500	0.833	X_4_ > X_1_ > X_5_ > X_2_ > X_3_
*C*_66_	1.000	1.000	0.833	0.500	1.000	X_4_ > X_3_ > X_1_ = X_2_ = X_3_
*C*_67_	1.000	0.805	1.000	1.000	0.500	X_5_ > X_2_ > X_1_ = X_3_ = X_4_

Note: We simply wrote P(X_i_ ≤ X_max_) as X_i_ in column “Priority”.

**Table 10:** Changes in all the criteria weights according to *C*_53_.

Criteria	BWM Weight	1	2	3	4	5	6	7	8	9
*C*_11_	0.051	0.049	0.044	0.038	0.033	0.027	0.022	0.016	0.011	0.005
*C*_12_	0.035	0.034	0.031	0.027	0.023	0.019	0.015	0.011	0.008	0.004
*C*_13_	0.028	0.027	0.024	0.021	0.018	0.015	0.012	0.009	0.006	0.003
*C*_14_	0.064	0.062	0.055	0.048	0.041	0.035	0.028	0.021	0.014	0.007
*C*_15_	0.014	0.014	0.012	0.011	0.009	0.008	0.006	0.005	0.003	0.002
*C*_21_	0.008	0.009	0.008	0.007	0.006	0.005	0.004	0.003	0.002	0.001
*C*_22_	0.021	0.020	0.018	0.016	0.014	0.011	0.009	0.007	0.005	0.002
*C*_23_	0.004	0.004	0.003	0.003	0.002	0.002	0.002	0.001	0.001	0.000
*C*_24_	0.006	0.006	0.005	0.005	0.004	0.003	0.003	0.002	0.001	0.001
*C*_25_	0.007	0.006	0.006	0.005	0.004	0.004	0.003	0.002	0.001	0.001
*C*_31_	0.070	0.068	0.060	0.053	0.045	0.038	0.030	0.023	0.015	0.008
*C*_32_	0.035	0.034	0.030	0.027	0.023	0.019	0.015	0.011	0.008	0.004
*C*_33_	0.011	0.010	0.009	0.008	0.007	0.006	0.005	0.003	0.002	0.001
*C*_34_	0.024	0.023	0.021	0.018	0.016	0.013	0.010	0.008	0.005	0.003
*C*_41_	0.055	0.053	0.047	0.041	0.035	0.030	0.024	0.018	0.012	0.006
*C*_42_	0.074	0.072	0.064	0.056	0.048	0.040	0.032	0.024	0.016	0.008
*C*_43_	0.036	0.035	0.031	0.027	0.023	0.020	0.016	0.012	0.008	0.004
*C*_44_	0.013	0.013	0.011	0.010	0.009	0.007	0.006	0.004	0.003	0.001
*C*_51_	0.014	0.014	0.012	0.011	0.009	0.008	0.006	0.005	0.003	0.002
*C*_52_	0.034	0.034	0.030	0.026	0.022	0.019	0.015	0.011	0.007	0.004
*C*_53_	0.075	0.100	0.200	0.300	0.400	0.500	0.600	0.700	0.800	0.900
*C*_54_	0.028	0.027	0.024	0.021	0.018	0.015	0.012	0.009	0.006	0.003
*C*_61_	0.070	0.068	0.061	0.053	0.046	0.038	0.030	0.023	0.015	0.008
*C*_62_	0.031	0.030	0.027	0.024	0.020	0.017	0.013	0.010	0.007	0.003
*C*_63_	0.060	0.059	0.052	0.046	0.039	0.033	0.026	0.020	0.013	0.007
*C*_64_	0.064	0.062	0.056	0.049	0.042	0.035	0.028	0.021	0.014	0.007
*C*_65_	0.010	0.010	0.008	0.007	0.006	0.005	0.004	0.003	0.002	0.001
*C*_66_	0.017	0.017	0.015	0.013	0.011	0.009	0.007	0.006	0.004	0.002
*C*_67_	0.040	0.039	0.035	0.031	0.026	0.022	0.017	0.013	0.009	0.004
Total	1	1	1	1	1	1	1	1	1	1

**Table 11:** Five urban environment quality ranking after 9 runs in the sensitivity analysis.

Cities	Normalized	Run1	Run2	Run2	Run3	Run4	Run5	Run6	Run7	Run8	Run9
X_1_	4	4	4	4	4	4	4	4	4	4	4
X_2_	5	5	5	5	5	5	5	5	5	5	5
X_3_	3	3	3	3	3	3	3	3	3	3	3
X_4_	1	1	1	1	1	1	1	1	1	1	1
X_5_	2	2	2	2	2	2	2	2	2	2	2

## Appendix A. Detailed Results

**Table A1:** Direct grey decision matrix *M*.

***C*_11_**		***C*_12_**		***C*_13_**		***C*_14_**		***C*_15_**		***C*_21_**		***C*_22_**		***C*_23_**		***C*_24_**		***C*_25_**		***C*_31_**		***C*_32_**		***C*_33_**		***C*_34_**		***C*_41_**
**L**	**U**	**L**	**U**	**L**	**U**	**L**	**U**	**L**	**U**	**L**	**U**	**L**	**U**	**L**	**U**	**L**	**U**	**L**	**U**	**L**	**U**	**L**	**U**	**L**	**U**	**L**	**U**	**L**
5.7	8.1	6	7.6	5.9	7.9	6.1	7.9	4.2	5.2	6	8.2	3.4	4.4	4	5.5	5	6.4	4.5	5.7	5.6	7.4	5.1	7.1	3.9	5	4.5	5.7	4.9
4.9	6.3	5.4	7	4.6	5.8	4.5	5.7	5.4	7.1	4.9	6.1	4.6	5.6	4.3	6.2	5.3	6.9	4.2	5.2	4.6	5.8	4.7	5.9	4.9	5.9	6.1	7.7	4.6
3.7	5.3	4.1	5.1	4.4	5.8	4.2	5.4	7	8.4	5.8	7.4	5.3	7.1	5.7	7.8	6.3	7.9	5.7	7.7	3.7	4.9	4.4	5.6	3.7	4.9	4.7	6.3	3.7
6.3	8.3	7.9	9.3	7.7	9.3	7.3	8.9	5.4	7.6	6.5	8.9	6.6	8.6	6.7	8.2	8	9.4	6.4	8	6.6	8.6	6.2	8.2	4.4	5.4	5.2	6.2	7.6
7.1	9.1	5.8	7.4	4.6	6.2	4.5	5.9	6.4	8.6	5.7	7.3	6	7.5	5.8	8	5.8	8	4.2	5.4	6.2	8	6.3	8.5	4.7	6.3	6.7	8.1	5
	***C*_42_**		***C*_43_**		***C*_44_**		***C*_51_**		***C*_52_**		***C*_53_**		***C*_54_**		***C*_61_**		***C*_62_**		***C*_63_**		***C*_64_**		***C*_65_**		***C*_66_**		***C*_67_**	
**U**	**L**	**U**	**L**	**U**	**L**	**U**	**L**	**U**	**L**	**U**	**L**	**U**	**L**	**U**	**L**	**U**	**L**	**U**	**L**	**U**	**L**	**U**	**L**	**U**	**L**	**U**	**L**	**U**
6.8	3.7	4.9	4.7	6.1	4.1	5.1	4.2	5.2	3.9	4.9	5.4	7.6	4.4	5.4	5	6.4	5.3	7.1	5.2	7	5.1	6.3	5.6	7.2	4.8	6.4	4.4	5.4
5.8	4.9	6.3	4.7	6.1	4.3	5.3	4.9	6.1	5.2	6.6	5.1	6.3	4.9	5.9	4.3	5.8	4.4	5.8	4.8	6.4	3.9	5.2	4.7	6.3	4.4	5.4	5	7.5
4.9	6.3	8.3	6.1	8.1	5.8	8	4.5	5.7	5.5	7.9	4.9	6.9	5.7	8.1	2.7	3.7	3.4	4.9	4.5	6.2	3.1	4.3	4.6	6.2	5.8	7	3.6	4.7
9	4.8	6	4.6	5.6	3.9	4.9	5.6	7.2	5.1	6.7	6.2	7.2	6.5	8.3	6.2	7.6	6.1	7.5	6.4	8	6.1	7.5	5.9	7.9	6.5	8.3	4.7	5.7
6.6	5.7	8.1	5.4	7.6	4.6	6.2	4.1	5.7	6.8	8.2	5.9	7.9	6.4	8.6	5	6.8	6	8.4	6.1	8.1	4.9	6.1	4.9	6.5	3.8	5	6.7	8.3

**Table A2:** Normalization of direct grey decision matrix *M**.

***C*_11_**		***C*_12_**		***C*_13_**		***C*_14_**		***C*_15_**		***C*_21_**		***C*_22_**		***C*_23_**		***C*_24_**		***C*_25_**		***C*_31_**		***C*_32_**		***C*_33_**		***C*_34_**		***C*_41_**
**L**	**U**	**L**	**U**	**L**	**U**	**L**	**U**	**L**	**U**	**L**	**U**	**L**	**U**	**L**	**U**	**L**	**U**	**L**	**U**	**L**	**U**	**L**	**U**	**L**	**U**	**L**	**U**	**L**
0.63	0.89	0.65	0.82	0.63	0.85	0.69	0.89	0.49	0.60	0.67	0.92	0.40	0.51	0.49	0.67	0.53	0.68	0.56	0.71	0.65	0.86	0.60	0.84	0.62	0.79	0.56	0.70	0.54
0.54	0.69	0.58	0.75	0.49	0.62	0.51	0.64	0.63	0.83	0.55	0.69	0.53	0.65	0.52	0.76	0.56	0.73	0.53	0.65	0.53	0.67	0.55	0.69	0.78	0.94	0.75	0.95	0.51
0.41	0.58	0.44	0.55	0.47	0.62	0.47	0.61	0.81	0.98	0.65	0.83	0.62	0.83	0.70	0.95	0.67	0.84	0.71	0.96	0.43	0.57	0.52	0.66	0.59	0.78	0.58	0.78	0.41
0.69	0.91	0.85	1.00	0.83	1.00	0.82	1.00	0.63	0.88	0.73	1.00	0.77	1.00	0.82	1.00	0.85	1.00	0.80	1.00	0.77	1.00	0.73	0.96	0.70	0.86	0.64	0.77	0.84
0.78	1.00	0.62	0.80	0.49	0.67	0.51	0.66	0.74	1.00	0.64	0.82	0.70	0.87	0.71	0.98	0.62	0.85	0.53	0.68	0.72	0.93	0.74	1.00	0.75	1.00	0.83	1.00	0.56
	***C*_42_**		***C*_43_**		***C*_44_**		***C*_51_**		***C*_52_**		***C*_53_**		***C*_54_**		***C*_61_**		***C*_62_**		***C*_63_**		***C*_64_**		***C*_65_**		***C*_66_**		***C*_67_**	
**U**	**L**	**U**	**L**	**U**	**L**	**U**	**L**	**U**	**L**	**U**	**L**	**U**	**L**	**U**	**L**	**U**	**L**	**U**	**L**	**U**	**L**	**U**	**L**	**U**	**L**	**U**	**L**	**U**
0.76	0.45	0.59	0.58	0.75	0.51	0.64	0.58	0.72	0.48	0.60	0.68	0.96	0.51	0.63	0.66	0.84	0.63	0.85	0.64	0.86	0.68	0.84	0.71	0.91	0.58	0.77	0.53	0.65
0.64	0.59	0.76	0.58	0.75	0.54	0.66	0.68	0.85	0.63	0.80	0.65	0.80	0.57	0.69	0.57	0.76	0.52	0.69	0.59	0.79	0.52	0.69	0.59	0.80	0.53	0.65	0.60	0.90
0.54	0.76	1.00	0.75	1.00	0.73	1.00	0.63	0.79	0.67	0.96	0.62	0.87	0.66	0.94	0.36	0.49	0.40	0.58	0.56	0.77	0.41	0.57	0.58	0.78	0.70	0.84	0.43	0.57
1.00	0.58	0.72	0.57	0.69	0.49	0.61	0.78	1.00	0.62	0.82	0.78	0.91	0.76	0.97	0.82	1.00	0.73	0.89	0.79	0.99	0.81	1.00	0.75	1.00	0.78	1.00	0.57	0.69
0.73	0.69	0.98	0.67	0.94	0.58	0.78	0.57	0.79	0.83	1.00	0.75	1.00	0.74	1.00	0.66	0.89	0.71	1.00	0.75	1.00	0.65	0.81	0.62	0.82	0.46	0.60	0.81	1.00

*M** means direct grey matrix.

**Table A3:** Weighted normalized grey relational matrix *M***.

***C*_11_**		***C*_12_**		***C*_13_**		***C*_14_**		***C*_15_**		***C*_21_**		***C*_22_**		***C*_23_**		***C*_24_**		***C*_25_**		***C*_31_**		***C*_32_**		***C*_33_**		***C*_34_**		***C*_41_**
**L**	**U**	**L**	**U**	**L**	**U**	**L**	**U**	**L**	**U**	**L**	**U**	**L**	**U**	**L**	**U**	**L**	**U**	**L**	**U**	**L**	**U**	**L**	**U**	**L**	**U**	**L**	**U**	**L**
0.032	0.045	0.023	0.029	0.093	0.125	0.228	0.295	0.036	0.044	0.006	0.008	0.008	0.011	0.002	0.003	0.003	0.004	0.004	0.005	0.045	0.060	0.021	0.029	0.007	0.008	0.013	0.017	0.030
0.027	0.035	0.021	0.027	0.073	0.092	0.168	0.213	0.046	0.060	0.005	0.006	0.011	0.014	0.002	0.003	0.003	0.005	0.003	0.004	0.037	0.047	0.019	0.024	0.008	0.010	0.018	0.023	0.028
0.021	0.029	0.016	0.019	0.070	0.092	0.157	0.202	0.059	0.071	0.006	0.007	0.013	0.017	0.003	0.004	0.004	0.005	0.005	0.006	0.030	0.040	0.018	0.023	0.006	0.008	0.014	0.019	0.022
0.035	0.046	0.030	0.035	0.122	0.147	0.273	0.333	0.046	0.064	0.006	0.009	0.016	0.021	0.003	0.004	0.005	0.006	0.005	0.007	0.054	0.070	0.026	0.034	0.007	0.009	0.015	0.018	0.046
0.039	0.051	0.022	0.028	0.073	0.098	0.168	0.220	0.054	0.073	0.006	0.007	0.015	0.018	0.003	0.004	0.004	0.005	0.003	0.004	0.050	0.065	0.026	0.035	0.008	0.011	0.020	0.024	0.030
	***C*_42_**		***C*_43_**		***C*_44_**		***C*_51_**		***C*_52_**		***C*_53_**		***C*_54_**		***C*_61_**		***C*_62_**		***C*_63_**		***C*_64_**		***C*_65_**		***C*_66_**		***C*_67_**	
**U**	**L**	**U**	**L**	**U**	**L**	**U**	**L**	**U**	**L**	**U**	**L**	**U**	**L**	**U**	**L**	**U**	**L**	**U**	**L**	**U**	**L**	**U**	**L**	**U**	**L**	**U**	**L**	**U**
0.041	0.033	0.044	0.021	0.027	0.007	0.008	0.008	0.010	0.016	0.021	0.051	0.072	0.014	0.018	0.046	0.059	0.020	0.026	0.039	0.052	0.044	0.054	0.007	0.009	0.010	0.013	0.021	0.026
0.035	0.044	0.056	0.021	0.027	0.007	0.009	0.010	0.012	0.022	0.028	0.048	0.060	0.016	0.019	0.040	0.054	0.016	0.021	0.036	0.048	0.033	0.045	0.006	0.008	0.009	0.011	0.024	0.036
0.030	0.056	0.074	0.027	0.036	0.010	0.013	0.009	0.011	0.023	0.033	0.046	0.065	0.019	0.026	0.025	0.034	0.013	0.018	0.033	0.046	0.027	0.037	0.006	0.008	0.012	0.014	0.017	0.023
0.055	0.043	0.053	0.021	0.025	0.006	0.008	0.011	0.014	0.021	0.028	0.059	0.068	0.021	0.027	0.057	0.070	0.023	0.028	0.048	0.059	0.052	0.064	0.007	0.010	0.013	0.017	0.023	0.028
0.040	0.051	0.072	0.024	0.034	0.008	0.010	0.008	0.011	0.029	0.034	0.056	0.075	0.021	0.028	0.046	0.063	0.022	0.031	0.045	0.060	0.042	0.052	0.006	0.008	0.008	0.010	0.033	0.040

*M*** means normalized grey relational matrix.
